# Depth of invasion in patients with early stage oral cancer staged by sentinel node biopsy

**DOI:** 10.1002/hed.25665

**Published:** 2019-01-28

**Authors:** Inne J. den Toom, Luuk M. Janssen, Robert J.J. van Es, K. Hakki Karagozoglu, Bart de Keizer, Stijn van Weert, Stefan M. Willems, Elisabeth Bloemena, C. René Leemans, Remco de Bree

**Affiliations:** ^1^ Department of Head and Neck Surgical Oncology, UMC Utrecht Cancer Center University Medical Center Utrecht Utrecht The Netherlands; ^2^ Department of Otolaryngology‐Head and Neck Surgery VU University Medical Center Amsterdam The Netherlands; ^3^ Department of Oral and Maxillofacial Surgery / Oral Pathology VU University Medical Center/Academic Center for Dentistry (ACTA) Amsterdam Amsterdam The Netherlands; ^4^ Department of Nuclear Medicine University Medical Center Utrecht Utrecht The Netherlands; ^5^ Department of Pathology University Medical Center Utrecht Utrecht The Netherlands; ^6^ Department of Pathology VU University Medical Center Amsterdam The Netherlands

**Keywords:** depth of invasion, lymph node metastases, Oral cancer, sentinel lymph node biopsy

## Abstract

**Background:**

To investigate if depth of invasion (DOI) can predict occult nodal disease in patients with cT1‐2N0 (7th TNM) oral squamous cell carcinoma (OSCC) staged by sentinel lymph node biopsy (SLNB).

**Methods:**

In 199 OSCC patients, DOI measurements and SLNB were performed.

**Results:**

Metastases were found in 64 of 199 patients (32%). Of these 64 patients, the mean DOI was 6.6 mm compared to 4.7 mm in patients without metastases (*P* = .003). The ROC‐curve showed an area under the curve of 0.65 with a most optimal cutoff point of 3.4 mm DOI (sensitivity 83% and specificity 47%). Regional metastases were found in 15% of patients with DOI ≤ 3.4 mm.

**Conclusion:**

DOI seems to be a poor predictor for regional metastasis in patients with cT1‐2N0 OSCC. Therefore, staging of the neck using SLNB in patients with early stage oral cancer should also be performed in tumors with limited DOI and probably in T3 (8th TNM) OSCC ≤4 cm diameter.

## 1 INTRODUCTION

In patients with oral squamous cell carcinoma (OSCC) presence of cervical metastases is regarded as the main prognostic factor.^1^, ^2^, ^3^, ^4^ More recently, sentinel lymph node biopsy (SLNB) in early stage oral cancer is gaining acceptance as a diagnostic staging method for occult regional metastasis. The most recent meta‐analysis found a pooled sensitivity of 87% (95% CI 85%‐89%), a negative predictive value of 94% (95% CI: 93%‐95%), and an AUC of 0.98 (95% CI: 0.97‐0.99).^5^ The SLNB procedure detected occult metastases in around 30% of the patients, who will be additionally treated with a complementary (selective) neck dissection or radiotherapy.^6^, ^7^

In case of elective neck dissection (END) as a histopathological staging method, depth of invasion (DOI) of the primary tumor is the most promising predictive factor for nodal metastases.^8^, ^9^, ^10^ Huang et al. performed a meta‐analysis and recommended END in case of tumor thickness of ≥4 mm.^8^ However, most of their included studies reported on DOI and used the definition according to Moore et al. to measure “from a theoretical reconstructed normal mucosal line to the deepest extent of growth.”^11^

The debate in literature is ongoing due to large variation in study groups, measurements techniques, and cutoff values.^9^ As reported in a recent large study, DOI was associated with a higher incidence of regional failure, but still has a poor sensitivity and specificity for nodal involvement.^12^ Brockhoff et al. found different DOI cutoff values for different tumor locations determining a 20% or greater risk of having nodal metastases. They suggested to offer a neck dissection at >2 mm DOI in tongue tumors, 2‐3 mm DOI in floor of mouth tumors and 3‐4 mm DOI for the retromolar trigone and alveolus/hard palate tumors.^13^

Several studies have been conducted to identify the best predictor for occult nodal disease in patients with early stage oral cancer.^4^, ^14^, ^15^, ^16^, ^17^, ^18^, ^19^, ^20^, ^21^, ^22^, ^23^ In most of studies, DOI turns out to be the best histopathological predictor for regional metastases.^4^, ^14^, ^15^, ^16^, ^17^, ^18^, ^19^, ^22^ This is also reflected in the new 8th TNM classification in which DOI is now incorporated as determinant for clinical and pathological T staging.^24^, ^25^, ^26^

SLNB allows us to histopathologically examine the lymph nodes with the highest risk of containing metastases more precise than routine examination of all lymph nodes in END.^27^ In SLNB‐negative patients, a watchful waiting strategy of the neck renders the opportunity for micrometastasis, which can easily missed by routine histopathological examination of a neck dissection specimen, to become clinical manifest.^28^ Therefore, SLNB can serve as a more accurate reference standard than END for the evaluation of tests predicting the presence of lymph node metastases.

The aim of this study was to assess if DOI of the primary tumor can predict occult nodal disease in patients with a cT1‐2N0 (according to 7th AJCC classification) OSCC who underwent SLNB.

## 2 PATIENTS AND METHODS

In two Dutch head and neck centers, 199 patients were prospectively enrolled between 2007 and 2016. All patients had early stage oral cancer, a clinically negative neck (cT1‐T2N0), underwent SLNB as staging method and were treated by means of transoral excision of the primary tumor.

Institutional approval was obtained. Written informed consent was not deemed necessary according to national medical ethical guidelines due to the retrospective nature of the study.

The SLNB procedure was performed according to the EANM/SENT joint practice guidelines as has been previously described.^6^, ^27^, ^29^

The sentinel lymph nodes (SLNs) were histopathologically examined by two experienced head and neck pathologists (SMW and EB). The SLNs were stained with hematoxylin‐eosin (H&E) and cytokeratin AE1/3 at step‐serial sectioning levels of 150 μm. At least 6 levels were investigated. Every sectioning level was also examined with additional keratin immunohistochemistry (IHC) and positive IHC slices were compared to H&E slices to confirm metastases.

For this study, patients with regional metastases during follow‐up in case of a negative SLNB (false‐negatives) were considered as patients positive for metastases.

**Figure 1:**
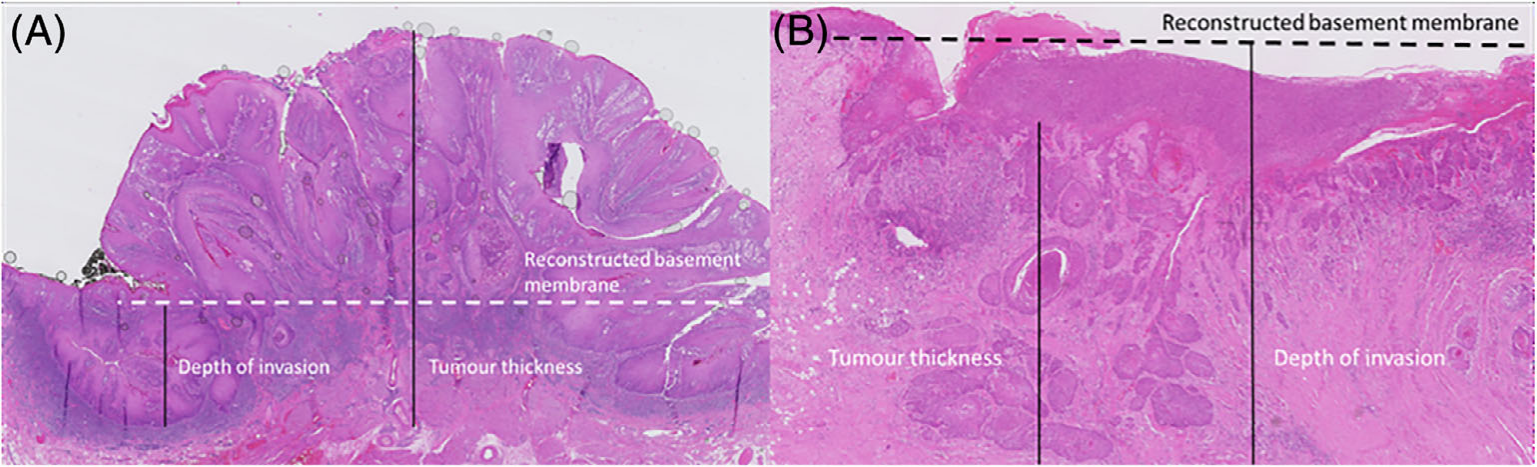
Measuring depth of invasion from the deepest point of invasion—reconstructed basement membrane line in exophytic tumor (A) and ulcerative tumor (B) [Color figure can be viewed at wileyonlinelibrary.com]

DOI of the primary tumor was measured by use of digital microscopic imaging or ocular micrometer. According to the 8th American Joint Committee on Cancer (AJCC) TNM classification, DOI was considered to be the actual mass present beneath the basement membrane, or in case of ulceration or exophytic lesions the theoretical reconstruction of the basement membrane (Figure 1).^24^

### 2.1 Statistical analysis

The Chi‐square test and Fisher exact test were used to compare categorical variables. DOI was correlated to nodal status with univariate logistic regression analysis. The receiver operating characteristic (ROC) curve was used to identify a possible cutoff value whereof DOI could serve as optimal predictor for regional metastases (and could act as deciding point performing a “watchful waiting” strategy or SLNB). All statistical analysis was carried out using SPSS 21 for Windows (IBM, Chicago, Illinois) in cooperation with a statistician.

### 2.2 8th American Joint Committee on Cancer TNM classification

The recent introduction of the 8th AJCC TNM classification system needs special attention because it specifically describes DOI as parameter in staging.^24^ The impact of using this system is described.^25^, ^26^

Tumors were staged according to both classifications and incidence of metastases according to T‐stage are presented. The 8th TNM classification is also used to see if a better distinction between T stages in overall survival, disease‐specific survival and isolated regional disease‐free survival could be made compared to the 7th TNM classification.

## 3 RESULTS

In this cohort of 199 cT1‐T2N0 patients at least one positive SLN was found in 52 (26%) patients. In another 12 patients with a (false) negative SLNB, regional metastases were encountered during follow‐up, which resulted in 64 (32%) patients with regional metastases. In these 64 cases, mean DOI was 6.6 mm (95% CI 5.48‐7.68) compared to 4.7 mm (95% CI 4.17‐5.21) in patients without regional metastasis (*P* = .003). Patient characteristics are listed in Table 1.

**Table 1:** Data of demographic and tumor‐related patient characteristics

Characteristics	Overall (%)	Histopathological status of the neck	
		Negative (%)	Positive (%)
Patients, No (%)	199 (100%)	135 (68%)	64 (32%)
Gender, No (%)			0
Male	100 (50%)	66 (66%)	34 (34%)
Female	99 (50%)	69 (70%)	30 (30%)
Median age (y) (range)	63 (27‐87)	64 (27‐87)	63 (29‐86)
Tumor location, No (%)			
Tongue	121 (61%)	80 (66%)	41 (34%)
Floor of mouth	53 (27%)	38 (72%)	15 (28%)
Buccal mucosa	16 (8%)	11 (69%)	5 (31%)
Inferior alveolar process	5 (3%)	3 (60%)	2 (40%)
Other	4 (2%)	3 (75%)	1 (25%)
Clinical T classification, No (%)^a^			
T1	132 (66%)	103 (78%)	029 (22%)
T2	67 (34%)	32 (48%)	35 (52%)
Depth of invasion, (mm) (95%CI)	5.3 (4.77‐5.81)	4.7 (4.17‐5.21)	6.6 (5.48‐7.68)
Follow‐up, (months) (range)			
Observation time	19 (1‐104)	20 (1‐104)	17 (1‐104)

a T classification according to 7th AJCC classification.

**Figure 2:**
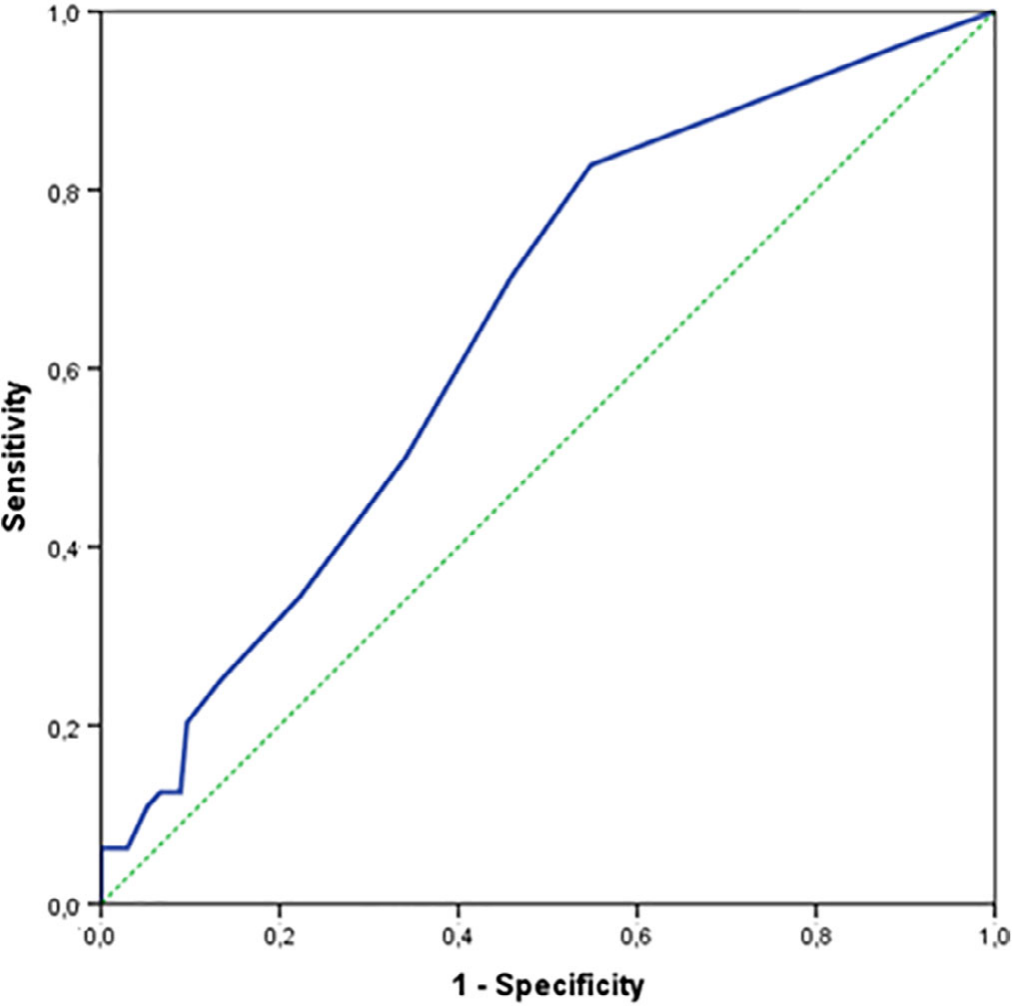
ROC‐curve for prediction of presence of lymph node metastasis by depth of invasion, area under the curve of 0.65 [Color figure can be viewed at wileyonlinelibrary.com]

**Figure 3:**
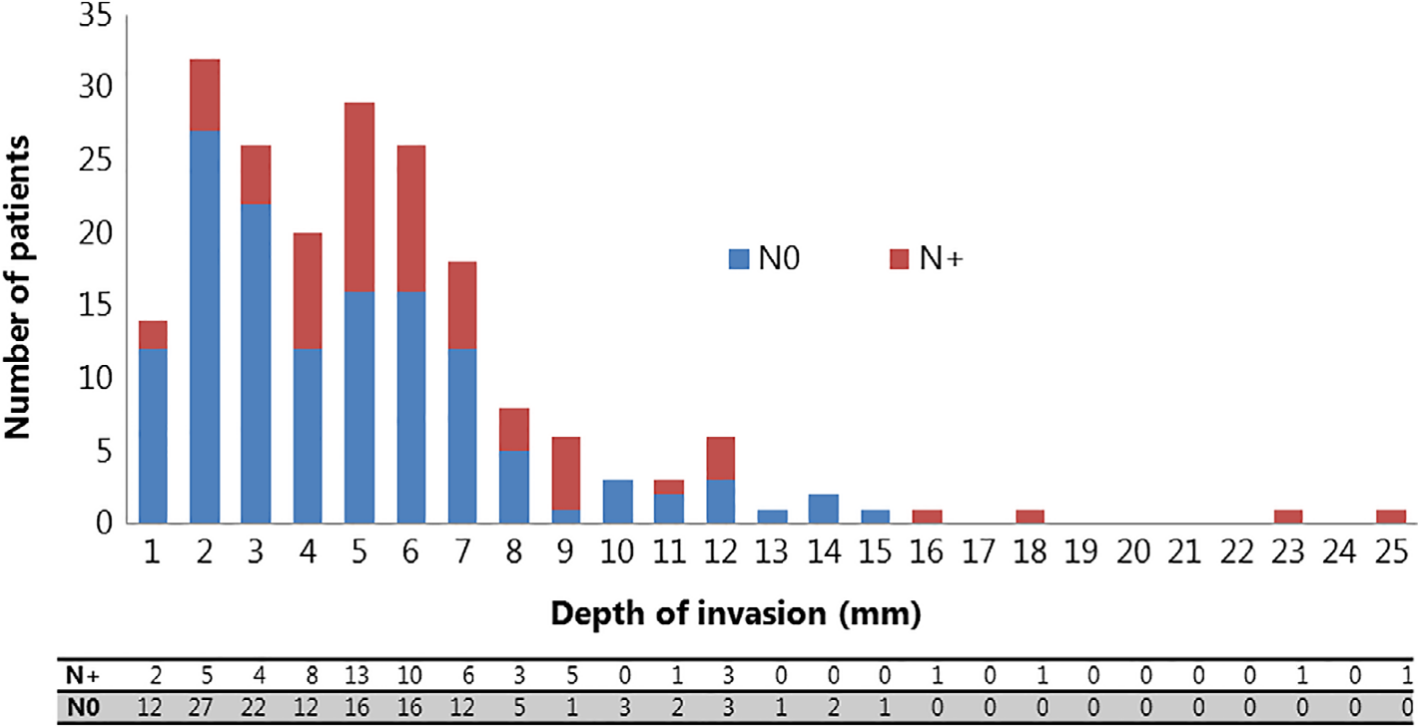
Distribution of nodal metastases per mm depth of invasion [Color figure can be viewed at wileyonlinelibrary.com]

In univariate logistic regression analysis an odds ratio of 1.15 (95% CI 1.05‐1.26) had been found for increasing DOI per 1 mm with a *P*‐value of .002. The ROC‐curve (Figure 2) showed an area under the curve of 0.65 with a most optimal cutoff point on a DOI of 3.4 mm (sensitivity 83%, specificity 47%) (Table 2). Of all patients with tumors ≤3.4 mm DOI, still 15% (11/74) had regional metastases, which is illustrated in Figure 3.

**Table 2:** Numbers for different cutoff values

DOI (mm)	Sensitivity	Specificity
1	97	9
2	89	29
3	83	45
4	70	54
5	50	66
6	34	78
7	25	87
8	20	90
9	13	91
10	13	93

### 3.1 8th American Joint Committee on Cancer TNM classification

The change in T‐classification (due to the influence of DOI as classification parameter) by using the new TNM staging system (8th edition) is listed in Table 3. In total, 49 pT1 tumors (TNM7) are classified as pT2 (TNM8) and 15 pT1 tumors (TNM7) are classified as pT3 tumors (TNM8).

**Table 3:** Shift in T stages according to 8th TNM classification

T stage	7th TNM	8th TNM	Upstaging
pT1	152 (76%)	88 (44%)	−64 (−42%)
pT2	44 (22%)	92 (46%)	+48 (+109%)
pT3	3 (2%)	19 (10%)	+16 (+533%)
Total	199 (100%)	199 (100%)	

**Figure 4:**
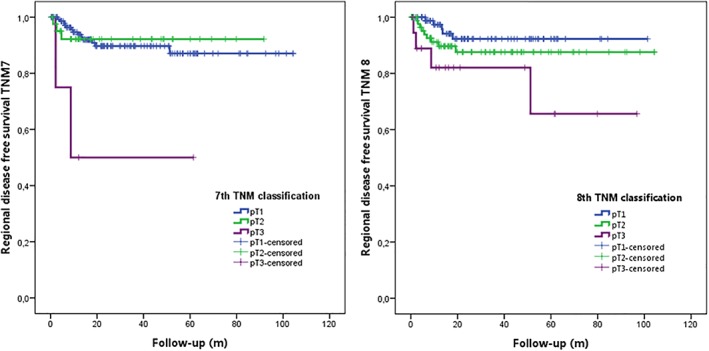
Isolated regional disease‐free survival analysis with 7th and 8th TNM classification respectively. Comparison between pT1 vs pT2 in 8th TNM classification did not reach statistical significance (*P* = .231) [Color figure can be viewed at wileyonlinelibrary.com]

No statistical significant difference between pT1 and pT2 tumors was found for isolated regional disease‐free survival (Figure 4), disease‐specific survival and overall survival using either the 7th or 8th edition of TNM classification. Because of the small numbers of pT3 tumors, no statistical analysis was performed comparing pT3 with pT1 and/or pT2 tumors.

Incidence of occult lymph node metastases according to pT classification was analyzed for both classifications and is listed in Table 4, showing in the 8th classification a decreased incidence in all T classifications, particularly for pT3 tumors.

**Table 4:** Incidence of occult lymph node metastases by T classification

T classification	7th TNM	8th TNM
pT1	40/152 (26%)	17/88 (19%)
pT2	21/44 (48%)	38/92 (41%)
pT3	3/3 (100%)	9/19 (47%)
Total	64/199 (32%)	64/199 (32%)

## 4 DISCUSSION

Based on our results, DOI could be considered as predictor for SLN metastases in early stage oral cancer. However, it should be clear that with an AUC of 0.65 in our ROC analysis the evidence for using it in clinical practice is at least questionable. Fifteen percent off all patients below the cutoff value of 3.4 mm had metastases, which makes it in our opinion reasonable to stage every patient with SLNB, regardless of DOI of the primary tumor.

With this study, we could identify metastases with use of the meticulous workup of SLNs by using step‐serial sectioning and additional keratin immunohistochemical staining, or in case of a negative SLNB by regular follow‐up. When comparing our results to the published literature some considerations have to be made.

First, it is essential to realize that the majority of published data about DOI and cervical lymph node metastases referred to END (or watchful waiting) as the gold standard.^4^, ^12^, ^14^, ^15^, ^17^, ^19^, ^22^, ^30^, ^31^, ^32^, ^33^, ^34^, ^35^, ^36^ To our knowledge, in only 3 articles, SLNB or SLNB‐assisted neck dissections were used as a staging tool.^18^, ^20^, ^37^ The routine histopathological workup of the END is less meticulous and hence presumably less accurate. Indeed, micrometastases remain undetected in up to 13% of routinely processed ENDs.^38^, ^39^ Using the SLNB protocol, the presence of metastasis can be determined more precisely within the lymph node with the highest risk (the SLN). Because of the “watchful waiting” strategy in case of a negative SLNB, isolated tumor cells and micrometastases can develop into a clinically detectable metastasis during follow‐up. Therefore, in our opinion, SLNB is a more accurate reference standard for staging the clinical negative neck than END.

Second, many studies have been published regarding this topic applying different definitions of DOI, infiltration depth, and tumor thickness. Originally described by Moore et al., DOI and tumor thickness are not the same.^11^ They performed a new measurement from an imaginary mucosal line (also defined in their article as a theoretical reconstruction of a basement membrane) besides the measurement of Breslow and they found a better correlation between survival and DOI by using this new line. This topic was later discussed in detail in the meta‐analysis of Pentenero et al. resulting in the recommendation to measure from the (theoretical reconstructed) basement membrane, which is also the recommendation of the AJCC.^9^, ^24^ It is essential to realize that measuring from the basement membrane is theoretically not the same as measuring from the mucosal line, which is mostly described as method for measuring the DOI. However, this is more for theoretical than practical purposes assuming the small thickness of healthy epithelium, so still reliable comparisons between both measurements could be made.

Although both meta‐analyses conclude that DOI correlates with regional lymph node involvement, they did mention different study groups, measurement techniques, and cutoff values, which hamper good comparison between these studies.^8^, ^9^ Both studies found a wide range for cutoff values of 1.5‐10 mm, with a most optimal cutoff value of 4 mm in the meta‐analysis of Huang et al.^8^ A recent large study of 469 patients, which was published after both meta‐analyses, used also a cutoff value of 4 mm to show an association between DOI and nodal involvement, though with poor sensitivity and specificity.^12^ The optimal cutoff value found in our study (3.4 mm) is close to this value. However, still 15% of our patients below this 3.4 mm cutoff value showed regional metastases. Therefore, in our opinion SLNB should be offered to all patients, also those with limited DOI tumors (Figure 3). Other studies using a ROC analysis to determine this optimal cutoff value found comparable values, that is, 4 mm and 4.59 mm.^5^, ^19^ The study of Goerkem et al. using this analysis did not found an optimal cutoff value.^20^ That study and our present one are the only studies that use SLNB‐alone as reference standard. In 78 patients, Goerkem et al. found an average DOI of 6.45 mm, with an area under the curve of 0.54 in the ROC analysis, concluding that DOI (and separately also tumor thickness) should not be used for assessment of elective treatment of the neck. Moreover, they suggested that SLNB should be used in all early stage oral cavity carcinomas with a cN0 neck.^20^

In another study, by Alkureishi et al., with SLNB (and SLNB‐assisted neck dissection) as reference standard a considerable heterogeneity in study groups has to be taken into account when comparing the results with the present study. In this study, patients with cT3‐T4 tumors and oropharyngeal tumors were included as well.^37^ They analyzed a cohort of 172 patients of whom 134 patients had oral tumors with a mean DOI of 7.3 mm. Patients underwent SLNB alone or SLNB‐assisted neck dissection, however the number of cases in both groups is unfortunately not reported. This may be important because histopathological examination of a neck dissection specimen is a suboptimal reference standard as compared to watchful waiting. They found nodal metastases in 41% of patients and demonstrated that in both oral and oropharyngeal cancer tumor depth reached a stronger correlation with nodal metastases than T‐classification. The most optimal cutoff value for oral cavity cancer alone in their cohort was 4 mm (sensitivity 83% and specificity 47%). Despite their higher mean DOI a comparable optimal cutoff value with comparable sensitivity and specificity rates has been found for oral cavity tumors only. They concluded that it is hard to predict which patients are at high risk for occult metastases based on a single tumor depth measurement.

In the article of Bilde et al., DOI (and tumor thickness) were significantly associated with cervical lymph node metastases with a cutoff value of 4 mm; however, no statistical analyses were presented for substantiation of this cutoff value.^18^ In addition, all patients were treated with SNB‐assisted neck dissection and the median tumor depth was 3.5 mm which makes an appropriate comparison with other studies difficult.

During the last years, DOI of the primary tumor is recognized to be of increasing value with respect to regional metastases and survival. A large international study demonstrated that using DOI with intervals of 5 mm improves discrimination in outcome.^40^ This is also reflected in the 8th TNM staging system by Amin et al. in which DOI, together with diameter of the tumor, classifies for T classification.^25^ With respect to our data, a large shift in pT classification was observed by using this new classification which is in agreement with other studies.^40^, ^41^, ^42^, ^43^ Interestingly, also the incidence numbers altered in the 8th classification (Table 4). In the pT3 (8th TNM classification) group, 47% of the patients showed regional metastases. In our opinion, these data suggest that SLNB could be helpful in patients with pT3cN0 OSCC ≤4 cm diameter, selecting more than half of them to avoid an unnecessary END.

Interestingly, with survival analyses for the 8th TNM classification slightly (but statistically not significant), better distinction was only observed in isolated regional disease‐free survival, while we expected to distinguish better in all survival analyses. Why we did not reach a evident correlation is hard to explain. Evidently, only early stage oral cancers were included. Possibly, pooling all T classifications, (T1‐T4) the new classification generally provides a better distinction compared to the 7th classification in our group of patients. These data have to be investigated in future research. In addition, this cohort is obviously smaller and with a shorter follow‐up in contrast to the previous analyses on which this new classification was based.^25^, ^40^ However, also Dirven et al. did not find a satisfying discrimination between pT1 and pT2 with respect to survival analyses in the 8th classification, although a comparison with the 7th classification was not established.^42^

Reliable clinical application of the TNM‐8 staging system is challenging. Most articles are based on specimen‐driven DOI measurements, whereas for pretreatment decision‐making DOI has to be clinically assessed. Lydiatt et al. describe that clinical examination of DOI requires careful palpation and attention to detail, supplemented by radiographic assessment.^26^

Recently, a meta‐analysis found a high correlation (*r* = 0.88) between intraoral ultrasonography and histopathological thickness measurements.^44^ Furthermore, Alsaffar et al. described a good correlation between clinical assessment, MRI, and pathology, particularly in thicker tumors.^45^ It should be clear that with the introduction of the 8th classification system, further research in preoperative measurements of DOI is required.

In conclusion, DOI seems to be a poor predictor for regional metastasis in patients with cT1‐2 N0 OSCC. Therefore, staging of the neck using SLNB in patients with early stage oral cancer should also be performed in tumors with limited DOI and probably in T3 (8th TNM) OSCC ≤4 cm diameter.

## CONFLICT OF INTEREST

The authors declare no conflict of interest.
